# Cell wall remodeling in mycorrhizal symbiosis: a way towards biotrophism

**DOI:** 10.3389/fpls.2014.00237

**Published:** 2014-06-04

**Authors:** Raffaella Balestrini, Paola Bonfante

**Affiliations:** ^1^Institute for Sustainable Plant Protection, National Research CouncilTorino, Italy; ^2^Department of Life Science and Systems Biology, University of TorinoTorino, Italy

**Keywords:** cell wall, mycorrhizal interactions, fungal genomes, interface, gene expression

## Abstract

Cell walls are deeply involved in the molecular talk between partners during plant and microbe interactions, and their role in mycorrhizae, i.e., the widespread symbiotic associations established between plant roots and soil fungi, has been investigated extensively. All mycorrhizal interactions achieve full symbiotic functionality through the development of an extensive contact surface between the plant and fungal cells, where signals and nutrients are exchanged. The exchange of molecules between the fungal and the plant cytoplasm takes place both through their plasma membranes and their cell walls; a functional compartment, known as the symbiotic interface, is thus defined. Among all the symbiotic interfaces, the complex intracellular interface of arbuscular mycorrhizal (AM) symbiosis has received a great deal of attention since its first description. Here, in fact, the host plasma membrane invaginates and proliferates around all the developing intracellular fungal structures, and cell wall material is laid down between this membrane and the fungal cell surface. By contrast, in ectomycorrhizae (ECM), where the fungus grows outside and between the root cells, plant and fungal cell walls are always in direct contact and form the interface between the two partners. The organization and composition of cell walls within the interface compartment is a topic that has attracted widespread attention, both in ecto- and endomycorrhizae. The aim of this review is to provide a general overview of the current knowledge on this topic by integrating morphological observations, which have illustrated cell wall features during mycorrhizal interactions, with the current data produced by genomic and transcriptomic approaches.

## INTRODUCTION

Plant cell walls form a dynamic extracellular matrix that actively controls growth and development, and are essential for the functionality of plants (Keegstra, 2010). Cell walls provide shape to the many different cell types needed to form tissues and organs and, forming the interface between neighboring cells, they control intercellular communication. Therefore, cell walls mediate most plant–microbe interactions. However, cell walls are not exclusive to photosynthetic organisms: fungi also have walls that determine hyphal growth, shape and responses (Durán and Nombela, 2004).

Unlike pathogenic interactions, where the fungal pathogen may be effective even with a limited presence in the plant tissues, in mycorrhizae, i.e., the widespread symbiotic associations established between plant roots and soil fungi (Bonfante and Genre, 2010), fungal colonization may involve as much as 80% of the secondary roots (Smith and Read, 2008). Irrespectively of their typology and their partner’s identity, mycorrhizal interactions achieve their functionality through the development of an extensive contact surface between plant and fungal cells, allowing signals and nutrients to be exchanged. In other words, the transfer of molecules from the fungus to the plant cytoplasm and *viceversa* takes place through both partners’ plasma membranes and cell walls, defining an apoplastic compartment known as the symbiotic interface on the basis of the first ultra-structural morphological observations (Scannerini and Bonfante-Fasolo, 1983).

In spite of the impressive biodiversity that is hidden behind the word “mycorrhiza” (Smith and Read, 2008), the interface has been considered a useful unifying concept to describe these plant-fungal interactions and to deal with both morphological (Bonfante, 2001; Peterson and Massicotte, 2004; Balestrini and Bonfante, 2005; Genre and Bonfante, 2012), molecular and genetic aspects (Harrison, 1999; Bücking et al., 2007; Reinhardt, 2007; Parniske, 2008; Gutjahr and Parniske, 2013).

The aim of this review is to provide an overview of the current knowledge on the dynamics of plant and fungal walls in mycorrhizae, as well as on their symbiotic interfaces, which – not surprisingly – have attracted a great deal of attention from the scientific community. Attention will mostly be focused on ectomycorrhizae (ECM) and arbuscular-mycorrhizae (AM). In ECMs the fungus covers the root tips, forming a mantle, and grows between the root cells, while in AM symbiosis the fungus develops inter- and intra-cellularly all along the root. Once the cortical layers are reached, fungal hyphae branch, leading to unique structures called arbuscules (Bonfante and Genre, 2010). The structural issues that result from morphological observations, and the biosynthetic aspects that stem from genomic and transcriptomic approaches, will be considered in this review.

## THE SYMBIOTIC INTERFACE: HOW TO INCREASE THE PARTNERS’ CONTACTS WHILE MAINTAINING BIOTROPHY

**FIGURE 1:**
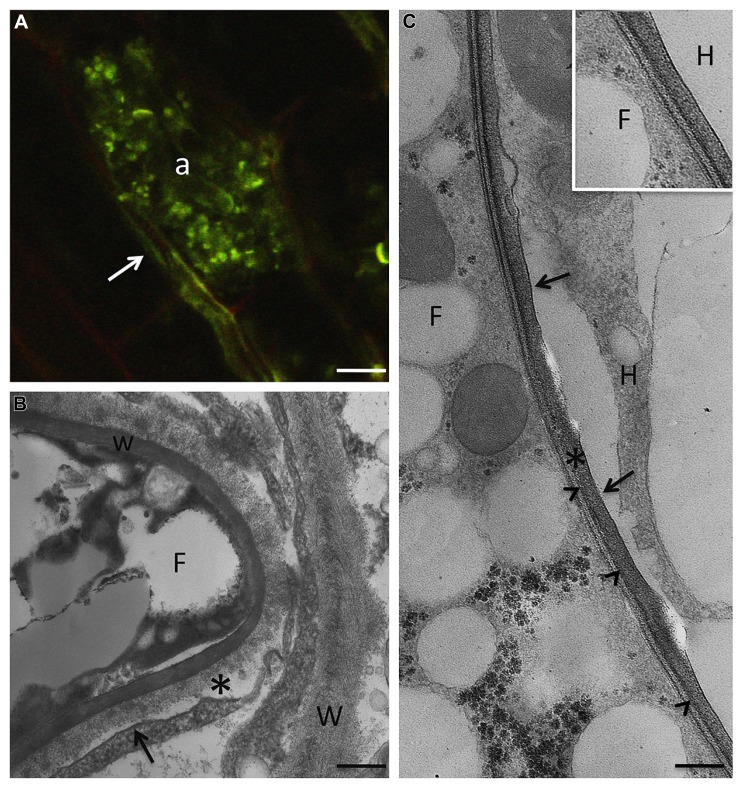
**In AM symbiosis, once the fungus overcomes the epidermal layer, it grows inter- and intracellularly all along the root in order to spread fungal structures.** Only when the fungus reaches the cortical layers, does a peculiar branching process that leads to the highly branched structures, called arbuscules, which are the main site for nutrient exchanges. **(A)** *R. irregularis* arbuscule (a) after staining with wheat germ agglutinin-FITC, on paraffin section of *M. truncatula* root, to detect chitin in the fungal cell wall. Arrow points to an intercellular hypha. Bar, 7 μm. **(B)** At the electron microscope level, a new apoplastic space, based on membrane proliferation (arrow), is evident around the intracellular hyphae (F). The picture shows the morphology of the interface material (asterisk), with respect to the plant cell walls (W), where neatly arranged fibers are evident. w, fungal cell wall. Bar, 0.17 μm. **(C)** The host membrane surrounding the fungus (F) is smooth (arrows) in a clover root prepared through high pressure/freeze substitution. The interface material is electron-dense after PATAg treatment, and the fungal wall (arrowheads) is very thin. Bar, 0.3 μm. Inset: High magnification of the interface compartment. F, fungus; H, host cell. Bar, 0.25 μm.

Among all the mycorrhizal interfaces, the complex intracellular interface of AM symbiosis has received considerable attention since its first descriptions in the seventies. Following the findings on fungal pathogens (Bracker and Littlefield, 1973; Scannerini and Bonfante, 1976) observed that the AM fungus is always surrounded by a plant-derived membrane, which leads to an interfacial zone consisting of a fungal plasma membrane, a specialized interfacial matrix, and a plant membrane, which was called the periarbuscular membrane (**Figure 1**). At that time, observations were mostly made on the cortical cells that host branched fungal arbuscules. The presence of this interface compartment is a typical feature of all endomycorrhizae (Scannerini and Bonfante-Fasolo, 1983; Peterson and Massicotte, 2004). In orchid, ericoid and arbutoid interactions, the intracellular fungus resulted to be confined within this compartment, that provides the structural basis of biotrophic interactions, since both partners maintain their individuality and remain alive. In the meantime, it causes a huge increase in the contact surface between the two partners, and the plant membrane increases in length several-fold during arbuscule development (Cox and Sanders, 1974).

The improved knowledge on the AM colonization process has allowed to demonstrate that the symbiotic interface is not limited to the arbusculated stage (Gutjahr and Parniske, 2013). When the AM germ-tube comes into contact with the epidermal cells, forming hyphopodia (Genre and Bonfante, 2007), these cells generate a colonization structure, the prepenetration apparatus (PPA), which is a transient formation that comprises cytoskeletal and endoplasmic reticulum (ER) components (Genre et al., 2005, 2008). Following this event, the hypha enters and crosses the epidermal cell, but prevents direct contact with the host cytoplasm thanks to a newly synthesized membrane of host origin. The biosynthesis of this novel perifungal membrane is the result of a process which involves exocytosis of the Golgi vesicles at the growing tips of the AM fungus. Using different GFP *Medicago* contructs, Genre et al. (2012) and Ivanov et al. (2012) have demonstrated that two VAMPs (vesicle-associated membrane proteins), belonging to R-SNAREs, are required to assemble the perifungal membrane. Interestingly, these proteins have also been detected at the cell plate of dividing meristematic root cells (Ivanov et al., 2012), thus offering the first molecular demonstration of the similarities between cell division leading to the construction of a new cell wall and development of the symbiotic interface.

Detailed electron microscope observations had already shown that the interfacial compartment contains cell-wall like material (Scannerini and Bonfante-Fasolo, 1983; Balestrini and Bonfante, 2005), and this has led to the question about whether the perifungal membrane maintains the capacity to synthesize and deliver cell wall-related molecules, like the peripheral membrane, (i.e., the plasma membrane of the host cell), but directed towards the interface space. The development of many *in situ* techniques (enzymes, lectins, and antibodies) has made it possible to validate this hypothesis: β-1,4-glucans, nonesterified homogalacturonans, xyloglucans, proteins rich in hydroxyproline (HRGPs), and arabinogalactan proteins (AGPs) have been located at the interface in many different plant/AM fungus combinations, as on peripheral cell wall, i.e., the cell wall of host cells containing the fungal structures (Bonfante et al., 1990a; Bonfante-Fasolo et al., 1991; Balestrini et al., 1994, 1996b; Gollotte et al., 1995; Balestrini and Bonfante, 2005). Expansins, which are extracellular proteins involved in cell wall-loosening and in the growth of plant cells (Cosgrove et al., 2002), have also been located in AM roots: they are present both in the cell walls of the host cells and in the interface, suggesting that this class of proteins, involved in cell wall loosening, may be crucial in the accommodation process of the fungus inside the cortical cells (Balestrini et al., 2005).

**FIGURE 2:**
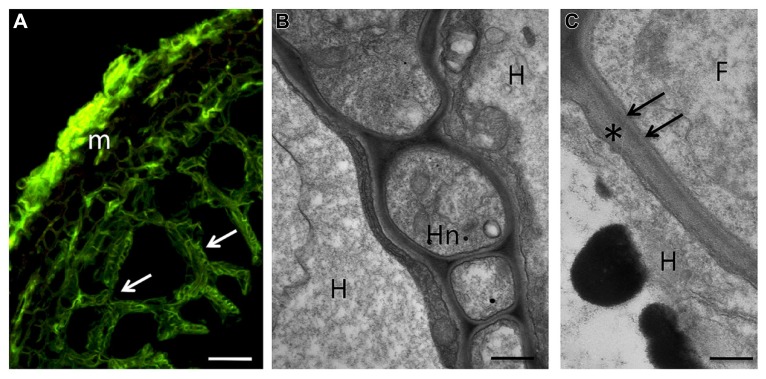
**During the symbiotic phase, ECM fungi form a fungal sheath (the mantle), which consists of aggregated hyphae that surround the root surface.** This mycelium is linked to extramatrical hyphae that explore the substrate and are responsible for the mineral nutrition and water uptake of the symbiotic tissues. Some hyphae from the inner zone of the mantle penetrate between the root cells to form the Hartig net, an intercellular hyphal network inside the root tissues where metabolites are exchanged between the symbiotic partners. The hyphae always remain apoplastic and can colonize epidermal and cortical cell layers. **(A)** Confocal micrograph showing a section of hazelnut – *Tuber melanosporum* ectomycorrhizal root. The mantle (m), formed by packed hyphae, and the Hartig net (arrows), which surrounds the epidermal and outer cortical cells, show a green signal after treatment with WGA-FITC. Bar, 15 μm. **(B)** Hartig net (Hn) in a fully truffle developed mycorrhiza. Hyphae develop among plant cells, and their cell walls are in direct contact with the plant cell walls, showing a simple interface structure. H, host cell; Hn, Hartig net. Bar, 0.6 μm. **(C)** Magnification of the contact zone between plant (asterisk) and fungal cell wall (arrows). F, fungus; H, host cell. Bar, 0.4 μm.

**FIGURE 3:**
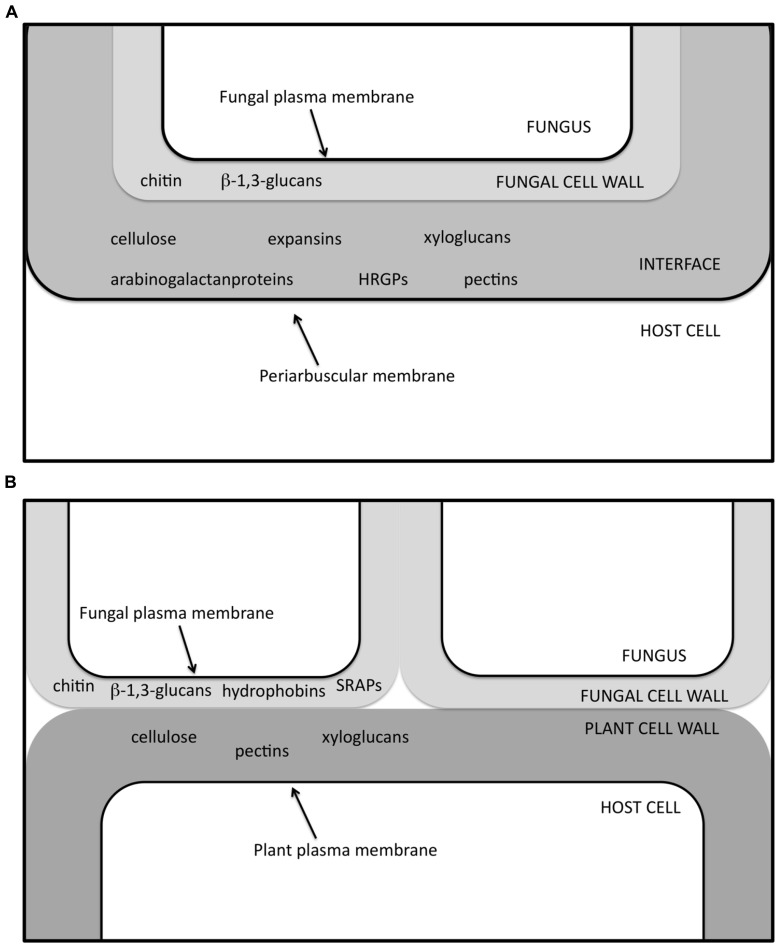
**Schematic view of the interface zone in AM (A) and ECM (B) symbiosis, in which several of the molecules so far determined through *in situ* labeling experiments (Balestrini et al., 1996a,b; Laurent et al., 1999; Tagu et al., 2001; Balestrini and Bonfante, 2005) are listed.** HRGPs, hydroxyproline-rich glycoprotein; SRAPs, symbiosis-regulated acidic polypeptides.

Although the molecular content of the interface reflects the composition of the host cell wall, the morphology of the interface material indicates that its components are assembled differently, and this leads to a more amorphous structure (**Figures 2** and **3**). In addition, this cell wall-like material changes in morphology during the arbuscule’s life cycle: it is very thick and compact around the arbuscule trunk, it becomes very thin around the fungal branches, and it again becomes thicker when the arbuscule collapses. Such dynamics probably mirror the uneven distribution of plant membrane proteins along the perifungal membrane (see Gutjahr and Parniske, 2013 for a review). Expansins and/or secreted proteins of fungal origin (see below) could also play a role in keeping the interfacial material loose.

In liverworts, the disappearance of cell wall autofluorescence, which is a normal feature of non-colonized parenchyma cells, has been observed in the fungus-colonized areas, suggesting changes in cell wall composition upon fungal colonization, and a localized decrease in cell wall-bound phenolic compounds (Ligrone et al., 2007).

Compared to AM symbiosis, the symbiotic interface in ECM appears much simpler, at least on a morphological level: the plant and fungal cell walls are always in direct contact, since the ECM fungus remains apoplastic (Balestrini et al., 1996a; Peterson et al., 2004; **Figures 2** and **3**). When the hyphae penetrate between the root cells, only subtle alterations can be observed in the PCW, although a localized loosening has been reported in several ECMs (Balestrini et al., 1996a). Ultra-structural observations of ECMs formed by *Hebeloma cylindrosporum* IAA-overproducing mutants suggest that fungal IAA may play a role in Hartig net development by affecting PCW loosening (Gea et al., 1994). Components specific to both plant (e.g., cellulose and pectins) and fungal (e.g., SRAPs and hydrophobins) cell walls have been identified in the cell walls at the symbiotic interface (Balestrini et al., 1996a; Laurent et al., 1999; Tagu et al., 2001; **Figure 3**).

In conclusion, functioning mycorrhizal symbiosis requires both partners to be alive in order to exchange nutrients in a balanced way (Kiers et al., 2011). From a structural point of view, this requires the partners to maintain their individuality: this is guaranteed by a simple wall-to-wall contact during intercellular interactions and by a more sophisticated interface when the fungus becomes intracellular. Interestingly, the development of such a complex interface is also characteristic of biotrophic pathogenic interactions (Yi and Valent, 2013), suggesting that some plant responses reflect ancient mechanisms which are independent of the outcome of the interaction. Quoting Yi and Valent (2013) “… the interface is the site of active secretion by both players. This cross-talk at the interface determines the winner in adversarial relationships and establishes the partnership in mutualistic relationships.”

## FROM STRUCTURE TO BIOSYNTHESIS: PLANT CELL WALL RELATED GENES RESPOND TO SYMBIOSIS

Starting from *in situ* localization data, attention has been focused on the genesis of the interfacial material, and in particular on the plant genes involved in the cell wall metabolism. Using targeted approaches, it was found that genes encoding a putative AGP and an HRGP were induced in mycorrhizal roots of *Medicago truncatula* and maize, respectively, and the transcripts were specifically localized in arbusculated cells (Balestrini et al., 1997; van Buuren et al., 1999). An endotransglucosylase/hydrolase gene, *Mt-XHT1*, induced in *M. truncatula* roots during AM symbiosis, was identified (Maldonado-Mendoza et al., 2005). The analysis of transgenic roots expressing an *Mt-XHT1* fusion promoter has shown that expression is enhanced not only in the root regions colonized by the fungus, but also at distal sites. On the basis of this expression pattern, it was suggested that Mt-XHT might be involved in the systemic modification of a cell wall structure in order to enable fungal penetration.

Coming to the availability of sequenced genomes for several plants that are AM hosts, and the possibility of obtaining global transcriptional profiling of mycorrhizal roots, the regulation of cell-wall related genes has been studied at a larger scale. On the basis of localization data, an expansin gene has been found among the most up-regulated genes in *M. truncatula* mycorrhizal roots (Liu et al., 2003). Similarly, expansin/expansin-like genes were up-regulated during the early symbiotic stages in both *M. truncatula* (Weidmann et al., 2004; Siciliano et al., 2007) and tomato roots (*EXLB1*; Dermatsev et al., 2009), suggesting that an increase in PCW plasticity is a prerequisite to the accommodation of the fungus in the plant. *In situ* hybridization experiments on *Medicago* roots have in fact revealed that an expansin-like protein was preferentially expressed in epidermal cells in contact with the hyphopodium (Siciliano et al., 2007). A cellulose synthase-like gene was also shown to be up-regulated at the same stage (Siciliano et al., 2007). In agreement with the proposed role played by the PPA, the up-regulation of genes involved in cell wall synthesis/remodeling, during hyphopodium development, suggests that the building of the interface starts prior to fungal colonization. The symbiosis-dependent expression of an early nodulation gene (*ENOD11),* induced during the early stages of root nodulation and coding for a putative proline-rich protein (PRP), has also been detected in epidermal and cortical cells during root colonization by AM fungi (Journet et al., 2001; Chabaud et al., 2002). However, the role of these PRP proteins remains purely hypothetical. Thanks to a cDNA microarray experiment on *Lotus japonicus*, Guether et al. (2009) have demonstrated that a large number of genes related to membrane dynamics and cell wall metabolism are induced in mycorrhizal roots. These data support the hypothesis of plant cells having an active role in fungus accommodation *via* membrane proliferation and cell wall construction. Among the up-regulated genes related to cell wall metabolism, transcripts encoding for an endo-β-1,4-beta-glucanase (*LjCel1*) and a putative cellulose synthase (*LjCesA*) were detected exclusively in arbusculated cells, using a microdissection approach. These data are in agreement with those previously observed for *M. truncatula,* where the homologous gene *MtCel1* was found to be specifically expressed during symbiosis and, more specifically, within arbusculated cells (Liu et al., 2003). Considering the membrane domain, it was suggested that MtCel1 was located in the periarbuscular membrane and that it was directly involved in the assembly of the cellulose/hemicellulose matrix detected at the interface through *in situ* methods. Similarly, transcripts of a putative cellulose synthase, *LjCesA*, were also found to accumulate in arbusculated cells. CesA proteins are part of the cellulose synthase complex in higher plants (Taylor, 2008), which is a membrane-located enzymatic system that is responsible for cellulose synthesis.

Morphometric analyses have suggested an increase in the size of cells containing arbuscules (Balestrini et al., 2005). The authors hypothesized that *LjCesA* plays a role in cell expansion during arbuscule development, in conjunction with other proteins involved in cell wall remodeling (Balestrini and Bonfante, 2005). The role of two apoplastic plant proteases of the subtilase family (LjSbtM1 and LjSbtM3) during AM fungal colonization was also reported (Takeda et al., 2009). The members inside this protease family, as extracellular enzymes, are expressed during organ development and may be involved in the modification of cell wall structure, thus contributing to cell wall dynamics (Schaller et al., 2012). It has been proposed that these two *Lotus* genes, which are specifically expressed during the symbiosis, could play a role in cell wall modifications: they could facilitate fungal growth, or communication between the symbiotic partners, e.g., through the generation of peptides with a signaling role. Localization data have also shown that LjSbtM1, which is targeted for secretion, is localized in the apoplastic compartments, including the PCWs of the colonized cells, the intercellular spaces and the periarbuscular space.

Considering these data, it is tempting to suggest that the AM fungus not only leads to the construction of the interface compartment, but may also have the peripheral cell wall as an additional target. This has also been suggested for an ascorbate oxidase gene (*LjAO1*), which is up-regulated during AM symbiosis, where it shows a double location: in the apoplast and in the interface. This suggests that AO is possibly involved in the accommodation of fungal structures, with the role of maintaining the structure of the interface compartment, as well as in cell wall loosening and extension, as observed in arbusculated cells (Balestrini et al., 2012a).

The activation of a β-xylosidase α-I-arabinosidase (which can contribute to the turnover of cell wall xylose and arabinose) has also been shown to be induced in tomato mycorrhizal roots (Fiorilli et al., 2009); the corresponding transcripts were localized exclusively in the arbuscule-containing cells, suggesting its involvement during the formation of the periarbuscular matrix, although involvement in the remodeling of the peripheral wall cannot be excluded. A monosaccharide transporter, MST2, which has been identified in an AM fungus (Helber et al., 2011), has been shown to transport not only glucose, but also cell wall monosaccharides, i.e., xylose. This result is in agreement with the fact that the plant-fungal interface in AM symbiosis contains primary PCW components (Balestrini and Bonfante, 2005). The authors have suggested that a versatile sugar transporter – capable of transporting monosaccharides from the apoplast – might be an optimal adaptation to the biotrophic life style of AMF (Helber et al., 2011).

Changes in the plant transcriptomic profiles of ECM roots have been well documented (Voiblet et al., 2001; Johansson et al., 2004; Duplessis et al., 2005; Le Quéré et al., 2005; Tuskan et al., 2006; Heller et al., 2008), although poplar is the only ECM-forming plant whose genome has been sequenced so far. As a consequence, the regulation of PCW genes has only been investigated poorly, while more information is available on the fungal side (see next section).

The regulation of some PCW-related genes, e.g., the down-regulation of a pectin methylesterase gene and the up-regulation of genes coding for an expansin and a cellulose-synthase-like, has been reported in *Paxillus involutus*-poplar ECMs (Luo et al., 2009). The peripheral root cells of these ECM plants are swollen in comparison to non-ECM root cells, suggesting a role of these genes in cell wall expansion that mirrors cell enlargement. Genes coding for PCW proteins, mainly PRP, have been found to be up-regulated during the ECM association between oak and *Piloderma croceum* (Tarkka et al., 2013). This result is in agreement with previous observations where one PRP transcript was over-expressed in pre-mycorrhizal and mature roots (Frettinger et al., 2007). Using an oak contig assembly, Tarkka et al. (2013) also revealed that two extensins and several peroxidase genes were down-regulated, suggesting a reduced potential for cross-linking of the cell wall components in ECMs, while an expansin contig, with a putative role in cell wall relaxation also resulted to be up-regulated.

In conclusion, the development of genomics and transcriptomics tools has made it possible to demonstrate that symbiotic interface construction leads to a consistent variation in the expression profile of many cell-wall related genes. In AMs, the up-regulation of genes linked to the synthesis of cellulose and other polysaccharides is in line with the presence of cell wall polysaccharides in the interfacial matrix. However, it is interesting to note that relevant transcriptomic changes are also detectable in the ECMs, where no conspicuous morphological modifications have been described. Finally, the impact of mycorrhizal fungi on the cell wall metabolism does not seem to be limited to the symbiotic interfaces, since a still not fully acknowledged aspect is the increase in size of the colonized cells, which likely results from cell wall relaxation. Interestingly, it has recently been demonstrated that a loss in function mutation in a mycorrhiza-specific maize *Pi* transporter gene (*Pht1;6*) leads to a down-regulation of cell wall-related genes in AM roots (Willmann et al., 2013), although the mechanisms at the base of this response still have to be investigated.

## DECIPHERING THE GENOMES OF MYCORRHIZAL FUNGI SHEDS LIGHT ON CELL WALL DYNAMICS

The sequenced genomes of mycorrhizal fungi are crucial tools to obtain a deeper understanding of the molecular mechanisms that underlie the symbiotic lifestyle. Focusing on cell wall genes, the so far sequenced fungi have allowed new information to be obtained on the fungal cell wall machinery, including genes that potentially code for fungal cell wall proteins involved in the interaction with plants, and those that may code for enzymes which act on PCW. This is a great step forward, since unlike for pathogenic fungi, a biochemical characterization of the main cell wall components, (i.e., polysaccharides and proteins) is not yet available for mycorrhizal fungi.

The involvement of fungal cell wall proteins, mainly small secreted proteins, (e.g., SRAPs and hydrophobins), has been well studied during the development of the symbiotic interface in ECM associations (Martin et al., 1999; Laurent et al., 1999; Tagu et al., 2001). Hydrophobins are morphogenetic small-secreted and moderately hydrophobic proteins that are typically present in fungi and which are involved in several aspects of fungal biology (Wösten, 2001). They are also thought to play important roles during ECM establishment (Tagu et al., 2001; Voiblet et al., 2001; Plett et al., 2012). They have been localized on the fungal cell wall in the symbiotic structures formed by *Pisolithus tinctorius* and *Eucalyptus globulus* (Tagu et al., 2001), and several hydrophobins are currently considered to belong to apoplastic MiSSPs (Mycorrhizal-induced Small Secreted Proteins; Plett et al., 2012). A genome-wide inventory of hydrophobin genes from two different genomes of the ECM fungus *L. bicolor* (Martin et al., 2008) has recently been obtained. This inventory shows a complex diversity and a range of expression profiles inside this multi-gene family (Plett et al., 2012). The authors suggest that, during evolution, some hydrophobin proteins might have acquired new roles that are specific of the mutualistic lifestyle. Hydrophobin genes have been identified in the *Tuber melanosporum* genome (Martin et al., 2010; Balestrini et al., 2012b), and, among these genes, *TmelHYD3,* seems to be slightly up-regulated in ECMs *vs* the free-living mycelium. However, these proteins seem to be absent in *Rhizophagus irregularis,* as in mildews where hydrophobins are lacking (Spanu et al., 2010).

The main carbohydrate components of fungal cell walls are chitin and glucans, and these have been localized in the cell walls of different mycorrhizal fungi (Bonfante et al., 1990b; Balestrini et al., 1994, 1996a, 2012b; Lemoine et al., 1995; **Figures 1** and **2**).

Taking advantage of the genome sequence, a genome-wide inventory of the proteins involved in cell wall synthesis and remodeling has been obtained for the *T. melanosporum* black truffle, while expression results have revealed that cell wall-related genes can be involved in the morphogenetic transition from mycelium growth to the ectomycorrhizal branched hyphae (Balestrini et al., 2012b).

In addition to their structural role, chitin-derived molecules are widespread microbial signals that trigger various defense responses in plant cells (Shimizu et al., 2010; Hayafune et al., 2014). In *Rhizobium-*legume symbiosis, the nitrogen-fixing bacteria produce lipochitooligosaccharides (LCOs), which are termed Nod factors (Dénarié and Cullimore, 1993). It has long been proposed that AM fungi also release signal molecules (Myc factors), which are essential for the recognition of the fungal partner (Catoira et al., 2000; Parniske, 2008). These molecular signals have recently been characterized as a mixture of sulfated and non-sulfated LCOs (Maillet et al., 2011). Myc-LCOs, which are present at very low concentrations in the exudates of mycorrhizal carrot roots and *R. irregularis* germinated spores, stimulate AM symbiosis formation, and increase root branching in *M. truncatula* (Maillet et al., 2011). Fungal orthologs of bacterial genes coding for enzymes involved in symbiotic LCO factors synthesis have not been identified so far in the genome of *R. irregularis*, the first AM fungus to be sequenced (Tisserant et al., 2013). Additionally, it has recently been observed that short-chain chitin oligomers (COs) of AM fungus origin activate a response (Ca^2^^+^ spiking) in root epidermal cells, with the maximum activity being observed for CO4 and CO5 (Genre et al., 2013). Short-chain COs are therefore part of the molecular dialog with the host plant, that leads to the activation of the common symbiosis signaling pathway: these data reveal that AM fungi may produce different chitin-based signals (Genre et al., 2013). The *R. irregularis* genome (Tisserant et al., 2013; Lin et al., 2014) has revealed many genes involved in the chitin metabolism. In addition, transcriptomic data have shown that several of these genes, including, e.g., chitin synthases and putative chitin deacetylases, are expressed during the different stages of the fungal life cycle, including the pre-symbiotic stage (Tisserant et al., 2012). Further investigation on the regulation of specific members inside these gene families, will provide new information on the relationships between the generation of chitin-derived signals by AM fungi and their life cycle. It is interesting to note that, until now, the production of chitin-related molecules with a signaling meaning has not been described in ECM fungi.

Fungal cell wall-related signals therefore seem to play an important role in pre-symbiotic communication between the AM symbiosis partners. This also seems to be true for the plant side: cutin monomers, which are hydrophobic components of the cell walls that are mostly present in epidermal cells, have been identified as signal molecules that induce hyphopodium differentiation (Wang et al., 2012; Murray et al., 2013).

Several fungal genes encoding enzymes involved in cell wall metabolism are expressed during AM fungal colonization, suggesting a role in the remodeling of the fungal cell wall during intracellular colonization (Lanfranco et al., 1999; Tisserant et al., 2012, 2013). AM fungal cell walls undergo a conspicuous change in their organization during their life cycle: the spore wall is thick and layered with a highly fibrillar chitin, while the hyphal wall becomes progressively thinner during the intracellular phase, reaching a thin amorphous structure in the thinner arbuscular branches (Bonfante et al., 1990b). This would seem to suggest that fungal growth inside the root cells requires a strict regulation of the genes related to both cell wall synthesis and degradation. Additionally, the expression of plant chitinase genes has been reported in arbuscule-containing cells, suggesting a putative role in fungal cell wall modification, during the development of the arbuscule branches, as well as in reducing the amount of chitin-derived elicitors, during the intracellular colonization and the development of the symbiotic interface (Bonanomi et al., 2001; Hogekamp et al., 2011).

As far as the *R. irregularis* genome is concerned, a surprising observation is the loss of glycosyl hydrolase (GH) genes and – among them – genes known to be involved in degrading PCW polysaccharides (Tisserant et al., 2013). PCW degradation requires the production of different enzymes that are regulated by the type and complexity of the plant material, and fungi can produce these enzymes (Tian et al., 2009). A decreased repertoire of PCW degrading enzymes, compared to saprotrophic and pathogenic fungi, has also been reported for the ECM fungi *L. bicolor* and *T. melanosporum* (Martin et al., 2008, 2010). Although both genomes have shown a reduced set of enzymes that target PCW components, subtle differences have been observed in the enzyme repertoire between the two fungi and in their expression (Martin et al., 2010; Plett and Martin, 2011). Despite the similarity of the symbiotic structures that they form, the expression results suggest differences in the mode of interaction with the respective host plants. For example, a limited number of PCW degrading enzymes are expressed in truffle symbiotic tissues, while *L. bicolor* expresses very few PCW degrading enzymes, and mainly secretes expansin-like proteins that may play a role in cell wall remodeling during hyphal penetration (Plett and Martin, 2011). Similarly, the ECM fungus *Amanita bisporigena* (Nagendran et al., 2009) lacks genes that code for extracellular enzymes which are active on cell wall linkages. A reduced set of PCW-degrading enzymes has also been found in some obligate biotrophic pathogens, i.e., *Blumeria graminis* (Spanu et al., 2010). Unlike the enzymatic arsenal observed in other pathogenic fungi, the great reduction in PCW-degrading enzymes in fungi that are phylogenetically and functionally diverse suggests that this feature is related to their biotrophic life style. This could be one of the strategies that biotrophic fungi adopt to lower the defense reactions of their hosts.

In conclusion, genome sequence projects have, for the first time, allowed hypotheses to be formulated on the meaning of biotrophy in mycorrhizal fungi. They may use chitin-related molecules to dialog with their plant partners, but in spite of their deep intra-root habit, they do not possess PCW-degrading enzymes that can act on the PCW. It can be hypothesized that the signal molecules released by mycorrhizal fungi are perceived by the plant cells, which – in turn – elicit the activation of their own PCW-degrading enzymes. This indirect mechanism should prevent the activation of strong defense reactions.

## CONCLUSION

Genome/transcriptome approaches applied to mycorrhizal fungi, and not only to the green partners, have had a profound effect on our knowledge of the biology of mycorrhizal symbiosis. As expected, the formation of the complex intracellular interface present in AMs is accompanied by a profound modulation of the PCW related genes (as well as the membrane-related ones). Surprisingly, important transcriptional changes have been detected in ECMs, where cell wall remodeling does not lead to evident morphological changes. The production of signaling molecules (Maillet et al., 2011; Genre et al., 2013), whose composition is related to the fungal cell wall, is probably a key element in the understanding of biotrophism. The mechanisms thanks to which the plant releases PCW enzymes, which allow fungal colonization, merit further detailed investigation. New genome data sets from several mycorrhizal fungi (ECM and AM), which are currently being sequenced (see http://genome.jgi.doe.gov/, http://mycor.nancy.inra.fr/genomeResources.html web sites), and from other non-AM endomycorrhizal fungi, e.g., *Oidiodendron maius* (ericoid symbiont) and *Tulasnella calospora* (orchid symbiont), will increase the knowledge on how different mycorrhizal fungi have an impact on PCW remodeling, and, consequently, on how the different mycorrhizal strategies have developed during the evolution.

## Conflict of Interest Statement

The authors declare that the research was conducted in the absence of any commercial or financial relationships that could be construed as a potential conflict of interest.

## References

[B1] Balestrini R. Hahn M. G. Bonfante P. (1996a). Location of cell-wall components in ectomycorrhizae of *Corylus avellana* and *Tuber magnatum*. *Protoplasma* 191 55–69 10.1007/BF01280825

[B2] Balestrini R. Hahn M. G. Faccio A. Mendgen K. Bonfante P. (1996b). Differential localization of carbohydrate epitopes in plant cell walls in the presence and absence of arbuscular mycorrhizal fungi. *Plant Physiol.* 111 203–213 10.1104/pp.111.1.203 12226286 PMC157827

[B3] Balestrini R. Romera C. Puigdomenech P. Bonfante P. (1994). Location of a cell-wall hydroxyproline-rich glycoprotein, cellulose and β-1,3-glucans in apical and differentiated regions of maize mycorrhizal roots. *Planta* 195 201–209 10.1007/BF00199680

[B4] Balestrini R. Jose-Estanyol M. Puigdomenech P. Bonfante P. (1997). Hydroxyproline-rich glycoprotein mRNA accumulation in maize root cells colonized by an arbuscular mycorrhizal fungus as revealed by *in situ* hybridization. *Protoplasma* 198 36–42 10.1007/BF01282129

[B5] Balestrini R. Bonfante P. (2005). The interface compartment in arbuscular mycorrhizae: a special type of plant cell wall? *Plant Biosyst.* 139 8–15 10.1080/11263500500056799

[B6] Balestrini R. Cosgrove D. J. Bonfante P. (2005). Differential location of α-expansin proteins during the accommodation of root cells to an arbuscular mycorrhizal fungus. *Planta* 220 889–899 10.1007/s00425-004-1431-1432 15605243

[B7] Balestrini R. Ott T. Guether M. Bonfante P. Udvardi M. K De Tullio M. C. (2012a). Ascorbate oxidase: the unexpected involvement of a ‘wasteful enzyme’ in the symbioses with nitrogen-fixing bacteria and arbuscular mycorrhizal fungi. *Plant Physiol. Biochem.* 59 71–79 10.1016/j.plaphy.2012.07.006 22863656

[B8] Balestrini R. Sillo F. Kohler A. Schneider G. Faccio A. Tisserant E. (2012b). Genome-wide analysis of cell wall-related genes in *Tuber melanosporum.* *Curr. Genet.* 58 165–177 10.1007/s00294-012-0374-6 22481122

[B9] Bonanomi A. Wiemken A. Boller T. Salzer P. (2001). Local induction of a mycorrhiza-specific class III chitinase gene in cortical root cells of *Medicago truncatula* containing developing or mature arbuscules. *Plant Biol.* 3 194–200 10.1055/s-2001-12902

[B10] Bonfante P. Vian B. Perotto S. Faccio A. Knox J. P. (1990a). Cellulose and pectin localization in roots of mycorrhizal *Allium porrum*: labeling continuity between host cell wall and interfacial material. *Planta* 180 537–547 10.1007/BF02411452 24202099

[B11] Bonfante P. Faccio A. Perotto S. Schubert A. (1990b). Correlation between chitin distribution and cell wall morphology in the mycorrhizal fungus *Glomus versiforme*. *Mycol. Res.* 94 157–165 10.1016/S0953-7562(09)80607-2

[B12] Bonfante-Fasolo P. Tamagnone L. Peretto R. Esquerré-Tugayé M. T. Mazau D. Mosiniak M. (1991). Immunocytochemical location of hydroxyproline rich glycoproteins at the interface between a mycorrhizal fungus and its host plants. *Protoplasma* 165 127–138 10.1007/BF01322283

[B13] Bonfante P. (2001). “At the interface between mycorrhizal fungi and plants: the structural organization of cell wall, plasma membrane and cytoskeleton,” in *The Mycota, IX: Fungal Associations* ed. Hock B. (Berlin: Springer) 45–61

[B14] Bonfante P. Genre A. (2010). Mechanisms underlying beneficial plant–fungus interactions in mycorrhizal symbiosis. *Nat. Commun.* 1 48 10.1038/ncomms1046 20975705

[B15] Bracker C. E. Littlefield L. J. (1973). “Structural concepts of host-pathogen interfaces,” in *Fungal Pathogenicity and the Plant’s Response* eds Byrde R. J. W. Cutting C. V. (London, NY: Academic Press) 159–317

[B16] Bücking H. Hans R. Heyser W. (2007). “The apoplast of ectomycorrhizal roots – site of nutrient uptake and nutrient exchange between the symbiotic partners,” in *The Apoplast of Higher Plants: Compartment of Storage, Transport and Reactions* eds Sattelmacher B. Horst W. J. (Dordrecht: Springer-Verlag) 97–108 10.1007/978-1-4020-5843-1_7

[B17] Catoira R. Galera C. de Billy F. Penmetsa R. V. Journet E. P. Maillet F. (2000). Four genes of *Medicago truncatula* controlling components of a nod factor transduction pathway. *Plant Cell* 12 1647–1666 10.1105/tpc.12.9.1647 11006338 PMC149076

[B18] Chabaud M. Venard C. Defaux-Petras A. Bécard G. Barker D. G. (2002). Targeted inoculation of *Medicago truncatula in vitro* root cultures reveals *MtENOD11* expression during early stages of infection by arbuscular mycorrhizal fungi. *New Phytol.* 156 265–273 10.1046/j.1469-8137.2002.00508.x 33873280

[B19] Cosgrove D. J. Li L. C. Cho H.-T. Hoffmann-Benning S. Moore R. C. Blecker D. (2002). The growing world of expansins. *Plant Cell Physiol.* 43 1436–1444 10.1093/pcp/pcf180 12514240

[B20] Cox G. Sanders F. (1974). Ultrastructure of the host-fungus interface in a vesicular-arbuscular mycorrhiza. *New Phytol.* 73 901–912 10.1111/j.1469-8137.1974.tb01319.x

[B21] Dermatsev V. Weingarten-Baror C. Resnick N. Gadkar V. Wininger S. Kolotilin I. (2009). Microarray analysis and functional tests suggest the involvement of expansins in the early stages of symbiosis of the arbuscular mycorrhizal fungus *Glomus intraradices* on tomato (*Solanum lycopersicum*). *Mol. Plant Pathol.* 11 121–135 10.1111/j.1364-3703.2009.00581.x 20078781 PMC6640415

[B22] Dénarié J. Cullimore J. (1993). Lipo-oligosaccharide nodulation factors: a new class of signalling molecules mediating recognition and morphogenesis. *Cell* 74 951–954 10.1016/0092-8674(93)90717-5 8402884

[B23] Duplessis S. Courty P. E. Tagu D. Martin F. (2005). Transcript patterns associated with ectomycorrhiza development in *Eucalyptus globulus* and *Pisolithus microcarpus*. *New Phytol.* 165 599–611 10.1111/j.1469-8137.2004.01248.x 15720670

[B24] Durán A. Nombela C. (2004). Fungal cell wall biogenesis: building a dynamic interface with the environment. *Microbiology* 150 3099–3103 10.1099/mic.0.27551-0 15470091

[B25] Fiorilli V. Catoni M. Miozzi L. Novero M. Accotto G. P. Lanfranco L. (2009). Global and cell-type gene expression profiles in tomato plants colonized by an arbuscular mycorrhizal fungus. *New Phytol.* 184 975–987 10.1111/j.1469-8137.2009.03031.x 19765230

[B26] Frettinger P. Derory J. Herrmann S. Plomion C. Lapeyrie F. Oelmüller R. (2007). Transcriptional changes in two types of pre-mycorrhizal roots and in ectomycorrhizas of oak microcuttings inoculated with *Piloderma croceum*. *Planta* 225 331–40 10.1007/s00425-006-0355-4 17016715

[B27] Gea L. Normand L. Vian B. Gay G. (1994). Structural aspects of ectomycorrhizas of Pinus pinaster (Ait.) Sol. formed by an IAA overproducer mutant of the fungus *Hebeloma cylindrosporum Romagnesi*. *New Phytol.* 128 659–670 10.1111/j.1469-8137.1994.tb04030.x

[B28] Genre A. Bonfante P. (2007). Check-in procedures for plant cell entry by biotrophic microbes. *Mol. Plant Microbe Interact.* 20 1023–1030 10.1094/MPMI-20-9-1023 17849704

[B29] Genre A. Bonfante P. (2012). “The interface between plants and mycorrhizal fungi: nutrient exchange, signaling and cell organization,” in *The Mycota, IX: Fungal Associations* 2nd Edn ed. Hock B. (Berlin: Springer) 39–49 10.1007/978-3-642-30826-0_3

[B30] Genre A. Chabaud M. Balzergue C. Puech-Pagès V. Novero M. Rey T. (2013). Short-chain chitin oligomers from arbuscular mycorrhizal fungi trigger nuclear Ca2+ spiking in *Medicago truncatula* roots and their production is enhanced by strigolactone. *New Phytol.* 198 190–202 10.1111/nph.12146 23384011

[B31] Genre A. Chabaud M. Faccio A. Barker D. G. Bonfante P. (2008). Prepenetration apparatus assembly precedes and predicts the colonization patterns of arbuscular mycorrhizal fungi within the root cortex of both *Medicago truncatula* and *Daucus carota*. *Plant Cell* 20 1407–1420 10.1105/tpc.108.059014 18515499 PMC2438458

[B32] Genre A. Chabaud M. Timmers T. Bonfante P. Barker D. G. (2005). Arbuscular mycorrhizal fungi elicit a novel intracellular apparatus in *Medicago truncatula* root epidermal cells before infection. *Plant Cell* 17 3489–3499 10.1105/tpc.105.035410 16284314 PMC1315383

[B33] Genre A. Ivanov S. Fendrych M. Faccio A. Žárský V. Bisseling T. (2012). Multiple exocytotic markers accumulate at the sites of perifungal membrane biogenesis in arbuscular mycorrhizas. *Plant Cell Physiol.* 53 244–255 10.1093/pcp/pBR170 22138099

[B34] Gollotte A. Gianinazzi-Pearson V. Gianinazzi S. (1995). Immunodetection of infection thread glycoprotein and arabinogalactan protein in wild type *Pisum sativum* (L.) or an isogenic mycorrhiza-resistant mutant interacting with *Glomus mosseae*. *Symbiosis* 18 69–85

[B35] Guether M. Balestrini R. Hannah M. He J. Udvardi M. K. Bonfante P. (2009). Genome-wide reprogramming of regulatory networks, transport, cell wall and membrane biogenesis during arbuscular mycorrhizal symbiosis in *Lotus japonicus*. *New Phytol.* 182 200–212 10.1111/j.1469-8137.2008.02725.x 19192192

[B36] Gutjahr C. Parniske M. (2013). Cell and developmental biology of arbuscular mycorrhiza symbiosis. *Annu. Rev. Cell Dev. Biol.* 29 593–617 10.1146/annurev-cellbio-101512-122413 24099088

[B37] Harrison M. J. (1999). Biotrophic interfaces and nutrient transport in plant fungal symbioses. *J. Exp. Bot.* 50 1013–1022 10.1093/jexbot/50.suppl_1.1013

[B38] Hayafune M. Berisio R. Marchetti R. Silipo A. Kayama M. Desaki Y. (2014). Chitin-induced activation of immune signaling by the rice receptor CEBiP relies on a unique sandwich-type dimerization. *Proc. Natl. Acad. Sci. U.S.A.* 111 E404–E413 10.1073/pnas.1312099111 24395781 PMC3903257

[B39] Helber N. Wippel K. Sauer N. Saarschmidt S. Hause B. Requena N. (2011). A versatile monosaccharide transporter that operates in the arbuscular mycorrhizal fungus *Glomus* sp. is crucial for the symbiotic relationship with plants. *Plant Cell* 23 3812–3823 10.1105/tpc.111.089813 21972259 PMC3229151

[B40] Heller G. Adomas A. Li G. Osborne J. van Zyl L. Sederoff R. (2008). Transcriptional analysis of *Pinus sylvestris* roots challenged with the ectomycorrhizal fungus *Laccaria bicolor*. *BMC Plant Biol.* 8:19. 10.1186/1471-2229-8-19 PMC2268937 18298811

[B41] Hogekamp C. Arndt D. Pereira P. A. Becker J. D. Hohnjec N. Küster H. (2011). Laser microdissection unravels cell-type-specific transcription in arbuscular mycorrhizal roots, including caat-box transcription factor gene expression correlating with fungal contact and spread. *Plant Physiol*. 157 2023–2043 10.1104/pp.111 22034628 PMC3327204

[B42] Keegstra K. (2010). Plant cell walls. *Plant Physiol.* 154 483–486 10.1104/pp.110.161240 20921169 PMC2949028

[B43] Kiers E. T. Duhamel M. Beesetty Y. Mensah J. A. Franken O. Verbruggen E. (2011). Reciprocal rewards stabilize cooperation in the mycorrhizal symbiosis. *Science* 333 880–882 10.1126/science.1208473 21836016

[B44] Ivanov S. Fedorova E. E. Limpens E. De Mita S. Genre A. Bonfante P. (2012). Rhizobium–legume symbiosis shares an exocytotic pathway required for arbuscule formation. *Proc. Natl. Acad. Sci. U.S.A.* 109 8316–8321 10.1073/pnas.1200407109 22566631 PMC3361388

[B45] Johansson T. Le Quéré A. Ahren D. Söderström B. Erlandsson R. Lundeberg J. (2004). Transcriptional responses of *Paxillus involutus* and *Betula pendula* during formation of ectomycorrhizal root tissue. *Mol. Plant Microbe Interact.* 17 202–215 10.1094/MPMI.2004.17.2.202 14964534

[B46] Journet E. P. El-Gachtouli N. Vernoud V. de Billy F. Pichon M. Dedieu A. (2001). *Medicago truncatula* ENOD11: a novel RPRPencoding early nodulin gene expressed during mycorrhization in arbuscule-containing cells. *Mol. Plant Microbe Interact.* 14 737–748 10.1094/MPMI.2001.14.6.737 11386369

[B47] Lanfranco L. Vallino M. Bonfante P. (1999). Expression of chitin synthase genes in the arbuscular mycorrhizal fungus *Gigaspora margarita*. *New Phytol.* 142 347–354 10.1046/j.1469-8137.1999.00393.x

[B48] Laurent P. Voiblet C. Tagu D. de Carvalho D. Nehls U. De Bellis R. (1999). A novel class of ectomycorrhiza-regulated cell wall polypeptides in *Pisolithus tinctorius*. *Mol. Plant Microbe Interact.* 12 862–871 10.1094/MPMI.1999.12.10.862 10517026

[B49] Lemoine M. C. Gollotte A. Gianinazzi-Pearson V. (1995). Localization of ß(1-3) glucan in walls of the endomycorrhizal fungi *Glomus mosseae* (Nicol. & Gerd.) Gerd. & Trappe and *Acaulospora laevis* Gerd. & Trappe during colonization of host roots. *New Phytol.* 129 97–105 10.1111/j.1469-8137.1995.tb03013.x 33874412

[B50] Le Quéré A. Wright D. P. Soderstrom B. Tunlid A. Johansson T. (2005). Global patterns of gene regulation associated with the development of ectomycorrhiza between birch (*Betula pendula* Roth.) and *Paxillus involutus* (Batsch) fr. *Mol. Plant Microbe Interact.* 18 659–673. 10.1094/MPMI-18-0659 16042012

[B51] Ligrone R. Carafa A. Lumini E. Bianciotto V. Bonfante P. Duckett J. G. (2007). Glomeromycotean associations in liverworts: A molecular cellular and taxonomic analysis. *Am. J. Bot.* 94 1756–1777 10.3732/ajb.94.11.1756 21636371

[B52] Lin K. Limpens E. Zhang Z. Ivanov S. Saunders D. G. O. Mu D. (2014). Single nucleus genome sequencing reveals high similarity among nuclei of an endomycorrhizal fungus. *PLoS Genet.* 10:e1004078. 10.1371/journal.pgen.1004078 PMC3886924 24415955

[B53] Liu J. Blaylock L. Endre G. Cho J. Town C. D. VandenBosch K. (2003). Transcript profiling coupled with spatial expression analyses reveals genes involved in distinct developmental stages of the arbuscular mycorrhizal symbiosis. *Plant Cell* 15 2106–2123 10.1105/tpc.014183 12953114 PMC181334

[B54] Luo Z.-B. Janz D. Jiang X. Göbel C. Wildhagen H. Tan Y. (2009). Upgrading root physiology for stress tolerance by ectomycorrhizas: Insights from metabolite and transcriptional profiling into reprogramming for stress anticipation. *Plant Physiol.* 151 1902–1917 10.1104/pp.109.143735 19812185 PMC2785981

[B55] Maillet F. Poinsot V. André O. Puech-Pagès V. Haouy A. Gueunier M. (2011). Fungal lipochitooligosaccharide symbiotic signals in arbuscular mycorrhiza. *Nature* 469 58–63 10.1038/nature09622 21209659

[B56] Maldonado-Mendoza I. E. Dewbre G. R. Blaylock L. Harrison M. J. (2005). Expression of a xyloglucan endotransglucosylase/hydrolase gene, Mt-XTH1, from *Medicago truncatula* is induced systemically in mycorrhizal roots. *Gene* 345 191197 10.1016/j.gene.2004.10.028 15716119

[B57] Martin F. Aerts A. Ahren D. Brun A. Danchin E. G. J. Duchaussoy F. (2008). Symbiosis insights from the genome of the mycorrhizal basidiomycete *Laccaria bicolor*. *Nature* 452 88–92 10.1038/nature06556 18322534

[B58] Martin F. Kohler A. Murat C. Balestrini R. Coutinho P. M. Jaillon O. (2010). Périgord black truffle genome uncovers evolutionary origins and mechanisms of symbiosis. *Nature* 464 1033–1038 10.1038/nature08867 20348908

[B59] Martin F. Laurent P. de Carvalho D. Voiblet C. Balestrini R. Bonfante P. (1999). Cell wall proteins of the ectomycorrhizal basidiomycete *Pisolithus tinctorius*: Identification, function, and expression in symbiosis. *Fungal Genet. Biol.* 27 161–174 10.1006/fgbi.1999.1138 10441442

[B60] Murray J. D. Cousins D. R. Jackson K. J. Liu C. (2013). Signaling at the root surface: The role of cutin monomers in mycorrhization. *Mol. Plant* 6 1381–1383 10.1093/mp/sst090 23935010 PMC3777838

[B61] Nagendran S. Hallen-Adams H. E. Paper J. M. Aslama N. Walton J. D. (2009). Reduced genomic potential for secreted plant cell-wall-degrading enzymes in the ectomycorrhizal fungus *Amanita bisporigera*, based on the secretome of *Trichoderma reesei*. *Fungal Genet. Biol.* 46 427–435 10.1016/j.fgb.2009.02.001 19373972

[B62] Parniske M. (2008). Arbuscular mycorrhiza: the mother of plant root endosymbioses. *Nat. Rev. Microb.* 6 763–775 10.1038/nrmicro1987 18794914

[B63] Peterson R. L. Massicotte H. B. (2004). Exploring structural definitions of mycorrhizas with emphasis on nutrient exchange interfaces. *Can. J. Bot.* 82 1074–1088 10.1139/b04-071

[B64] Peterson R. L. Massicotte H. B. Melville L. H. (2004). *Mycorrhizas: Anatomy and Cell Biology*. Toronto: CABI

[B65] Plett J. M. Gibon J. Kohler A. Duffy K. Hoegger P. J. Velagapudi R. (2012). Phylogenetic, genomic organization and expression analysis of hydrophobin genes in the ectomycorrhizal basidiomycete *Laccaria bicolor*. *Fungal Genet. Biol.* 49 199–209 10.1016/j.fgb.2012.01.002 22293303

[B66] Plett J. M. Martin F. (2011). Blurred boundaries: lifestyle lessons from ectomycorrhizal fungal genomes. *Trends Genet.* 27 14–22 10.1016/j.tig.2010.10.005 21112661

[B67] Reinhardt D. (2007). Programming good relations – development of the arbuscular mycorrhizal symbiosis. *Curr. Opin. Plant Biol.* 10 98–105 10.1016/j.pbi.2006.11.001 17127091

[B68] Scannerini S. Bonfante P. (1976). “Ultrastructural features of a vesicular-arbuscular mycorrhiza,” in *Sixth European Congress on Electron Microscopy* Vol. 2 ed. Ben-Shaul Y. (Jerusalem: TAL Ramat Gan.) 492–494

[B69] Scannerini S. Bonfante-Fasolo P. (1983). Comparative ultrastructural analysis of mycorrhizal associations. *Can. J. Bot.* 61 917–943 10.1139/b83-104

[B70] Schaller A. Stintzi A. Graff L. (2012). Subtilases – versatile tools for protein turnover, plant development, and interactions with the environment. *Physiol. Plant.* 145 52–66 10.1111/j.1399-3054.2011.01529.x 21988125

[B71] Shimizu T. Nakano T. Takamizawa D. Desaki Y. Ishii-Minami N. Nishizawa Y. (2010). Two LysM receptor molecules, CEBiP and OsCERK1, cooperatively regulate chitin elicitor signaling in rice. *Plant J.* 64 204–214 10.1111/j.1365-313X.2010.04324.x 21070404 PMC2996852

[B72] Siciliano V. Genre A. Balestrini R. Cappellazzo G. deWit P. J. G. M. Bonfante P. (2007). Transcriptome analysis of arbuscular mycorrhizal roots during development of the prepenetration apparatus. *Plant Physiol.* 144 1455–1466 10.1104/pp.107 17468219 PMC1914140

[B73] Smith S. E. Read D. J. (2008). *Mycorrhizal Symbiosis*, 3rd Edn. London: Academic Press

[B74] Spanu P. D. Abbott J. C. Amselem J. Burgis T. A. Soanes D. M. Stüber K. (2010). Genome expansion and gene loss in powdery mildew fungi reveal functional tradeoffs in extreme parasitism. *Science* 330 1543–1546 10.1126/science.1194573 21148392

[B75] Tagu D. De Bellis R. Balestrini R. De Vries O. M. H. Piccoli G. Stocchi V. (2001). Immunolocalization of hydrophobin HYDPt-1 from the ectomycorrhizal basidiomycete *Pisolithus tinctorius* during colonization of *Eucalyptus globulus* roots. *New Phytol.* 149 127–135 10.1046/j.1469-8137.2001.00009.x 33853243

[B76] Takeda N. Sato S. Asamizu E. Tabata S. Parniske M. (2009). Apoplastic plant subtilases support arbuscular mycorrhiza development in *Lotus japonicus*. *Plant J.* 58 766–777 10.1111/j.1365-313X.2009.03824.x 19220794

[B77] Tarkka M. T. Herrmann S. Wubet T. Feldhahn L. Recht S. Kurth F. (2013). OakContigDF159.1, A reference library for studying differential gene expression in *Quercus robur* during controlled biotic interactions: use for quantitative transcriptomic profiling of oak roots in ectomycorrhizal symbiosis. *New Phytol.* 199 529–540. 10.1111/nph.12317 23672230

[B78] Taylor N. G. (2008). Cellulose biosynthesis and deposition in higher plants. *New Phytol.* 178 239–252 10.1111/j.1469-8137.2008.02385.x 18298430

[B79] Tian C. Beeson W. T. Iavarone A. T. Sun J. Marletta M. A. Cate J. H. D. (2009). Systems analysis of plant cell wall degradation by the model filamentous fungus *Neurospora crassa*. *Proc. Natl. Acad. Sci. U.S.A.* 106 22157–22162 10.1073/pnas.0906810106 20018766 PMC2794032

[B80] Tisserant E. Kohler A. Dozolme-Seddas P. Balestrini R. Benabdellah K. Colard A. (2012). The transcriptome of the arbuscular mycorrhizal fungus *Glomus intraradices* (DAOM 197198) reveals functional tradeoffs in an obligate symbiont. *New Phytol.* 193 755–769 10.1111/j.1469-8137.2011.03948.x 22092242

[B81] Tisserant E. Malbreil M. Kuo A. Kohler A. Symeonidi A. Balestrini R. (2013). Genome of an arbuscular mycorrhizal fungus provides insight into the oldest plant symbiosis. *Proc. Natl. Acad. Sci. U.S.A.* 110 20117–20122 10.1073/pnas.1313452110 24277808 PMC3864322

[B82] Tuskan G. A. Difazio S. Jansson S. Bohlmann J. Grigoriev I. Hellsten U. (2006). The genome of black cottonwood, *Populus trichocarpa* (Torr. & Gray). *Science* 313 1596–604 10.1126/science.1128691 16973872

[B83] van Buuren M. L. Maldonado-Mendosa I. E. Trieu A. T. Blaylock L. A. Harrison M. J. (1999). Novel genes induced during an arbuscular mycorrhizal symbiosis formed between *Medicago truncatula* and *Glomus versiforme*. *Mol. Plant Microbe Interact.* 12 171–181 10.1094/MPMI.1999.12.3.171 10065555

[B84] Voiblet C. Duplessis S. Encelot N. Martin F. (2001). Identification of symbiosis-regulated genes in *Eucalyptus globulus–Pisolithus tinctorius ectomycorrhiza* by differential hybridization of arrayed cDNAs. *Plant J.* 25 181–191 10.1111/j.1365-313X.2001.00953.x 11169194

[B85] Wang E. Schornack S. Marsh J. F. Gobbato E. Schwessinger B. Eastmond P. (2012). A common signaling process that promotes mycorrhizal and oomycete colonization of plants. *Curr. Biol.* 22 2242–2246 10.1016/j.cub.2012.09.043 23122843

[B86] Weidmann S. Sanchez L. Descombin J. Chatagnier O. Gianinazzi S. Gianinazzi-Pearson V. (2004). Fungal elicitation of signal transduction related plant genes precedes mycorrhiza establishment and requires the dmi3 gene in *Medicago truncatula*. *Mol. Plant Microbe Interact.* 17 1385–1393 10.1094/MPMI.2004.17.12.1385 15597744

[B87] Willmann M. Gerlach N. Buer B. Polatajko A. Nagy R. Koebke E. (2013). Mycorrhizal phosphate uptake pathway in maize: vital for growth and cob development on nutrient poor agricultural and greenhouse soils. *Front. Plant Sci.* 4:533. 10.3389/fpls.2013.00533 PMC3872827 24409191

[B88] Wösten H. (2001). Hydrophobins: multipurpose proteins. *Annu. Rev. Microbiol*. 55 625–646 10.1146/annurev.micro.55.1.625 11544369

[B89] Yi M. Valent B. (2013). Communication between filamentous pathogens and plants at the biotrophic interface. *Annu. Rev. Phytopathol.* 51 587–611 10.1146/annurev-phyto-081211-172916 23750888

